# Unsupervised Machine Learning for Developing Personalised Behaviour Models Using Activity Data

**DOI:** 10.3390/s17051034

**Published:** 2017-05-04

**Authors:** Laura Fiorini, Filippo Cavallo, Paolo Dario, Alexandra Eavis, Praminda Caleb-Solly

**Affiliations:** 1 The BioRobotics Institute, Scuola Superiore Sant’Anna, Pontedera, Pisa 56025, Italy; filippo.cavallo@santannapisa.it (F.C.); paolo.dario@santannapisa.it (P.D.); 2 Alcove Limited, 44 Westbridge Road, London SW11 3PW, UK; alexandra.eavis@youralcove.com; 3 Bristol Robotics Laboratory, University of West England, Bristol BS16 1QY, UK; Praminda.Caleb-solly@uwe.ac.uk

**Keywords:** behavioural models, unsupervised machine learning, cognitive health assessment, real-home settings

## Abstract

The goal of this study is to address two major issues that undermine the large scale deployment of smart home sensing solutions in people’s homes. These include the costs associated with having to install and maintain a large number of sensors, and the pragmatics of annotating numerous sensor data streams for activity classification. Our aim was therefore to propose a method to describe individual users’ behavioural patterns starting from unannotated data analysis of a minimal number of sensors and a ”blind” approach for activity recognition. The methodology included processing and analysing sensor data from 17 older adults living in community-based housing to extract activity information at different times of the day. The findings illustrate that 55 days of sensor data from a sensor configuration comprising three sensors, and extracting appropriate features including a “busyness” measure, are adequate to build robust models which can be used for clustering individuals based on their behaviour patterns with a high degree of accuracy (>85%). The obtained clusters can be used to describe individual behaviour over different times of the day. This approach suggests a scalable solution to support optimising the personalisation of care by utilising low-cost sensing and analysis. This approach could be used to track a person’s needs over time and fine-tune their care plan on an ongoing basis in a cost-effective manner.

## 1. Introduction

Many older adults prefer to live independently in their home for as long as possible, but sometimes this is not possible because of the reduction in quality of life due to age-related decline resulting in cognitive and physical impairments and frailty, which can also cause safely concerns if they are on their own [1]. A significant proportion of older adults suffer from cognitive disorders such as Alzheimer’s disease and dementia. The European Union considers dementia as one of the most important causes of cognitive disorders and disability which affects the older population. In particular, it has been projected that there will be 7.7 million new cases of dementia each year [2]. In this context, researchers in the Ambient Assisted Living (AAL) field try to find cost-effective solutions to enhance independent living and promote active aging. Gustavsson et al. [3] estimate that the cost of the informal care provided to dementia suffers is around 72.5 billion euros across Europe. In this context, any technological tool which can assist these frail people on a daily basis would help to reduce costs [4,5].

Several clinical studies report a correlation between an older person’s health status and their daily behaviour [6]. For instance, the effect of ageing could include changes in sleep patterns. Sometimes people over 75 experience fragmentation of night-time sleep [7], whereas individuals with cognitive disorders could present problems related to the continuity of the sleep-awake cycle [8,9]. Other research demonstrates a close connection between brain function and the walking speed of older adults [10]. Additionally, other clinical studies have also demonstrated that individuals with cognitive impairments have difficulties in completing everyday activities when compared with healthy controls [11,12].

The recent advances in Internet of Things (IoT) and ambient intelligent solutions has led to a rapid increase in smart home technology. These smart and robotic systems can automatically collect information about users’ everyday habits [13]. For example, Information and Communications Technology (ICT) solutions can be used to investigate sedentary versus active behavior, and nights of restful sleep versus wandering and more disturbed nights. Consequently, if technology and reasoning could help to distinguish behavioural patterns, any anomalous events indicating changes in behavioural patterns could also be detected.

However, in order to realise the potential of IoT and AAL solutions in the real-world, there are a number of aspects that need to be addressed. Firstly, the IoT system should include a minimum number of unobtrusive sensors [14] to ensure user acceptance, as well as easy and effective installation and maintenance of the sensors. Often, these kinds of systems use cameras which are considered too invasive from the end-user’s perspective [15]. Additionally, pattern learning algorithms should be able to operate without labelled data, as it is too resource intensive for someone to manually verify the large quantities of data generated in a real living environment [16]. This last aspect is not straightforward due to the complexity of human behaviour.

Another major issue relates to the economics of optimal service provision. Care providers will not use these technological tools in their work if they are not cost-effective or do not provide significant benefits [17]. Carers work under time-pressure and they need to maximise their effectiveness in the time available. As such, they need to be able to prioritise a person’s needs and provide services to the best possible standard with the available resources. Optimal scheduling is a challenge and there are different approaches used to address timing and task constraints [18]. As such, providing carers with more information regarding people’s patterns and routines can help them take user preferences and needs into consideration and thus provide a more personalised and responsive service. In this sense, identifying the optimal data visualization strategy and clustering large volumes of data could support the carers in this task.

This work is based on unsupervised machine learning algorithms to discover potential behaviour-related features from low-level sensors that can be easily installed in the home. In this paper, we present our work on pattern recognition for developing personalised behavioural models and visualisation of unlabelled Passive InfraRed (PIR) motion sensor data with a dissimilarity analysis based on a distance measure in the feature space. In particular, our findings illustrate the efficacy of using a reduced number of commercial off-the shelf sensors to define a set of features for recognising users’ behaviour over a short learning period from unlabelled data.

Moreover, in this study a “blind” approach was proposed. This meant that all the analysis was conducted in an anonymised manner, without any a priori knowledge of personal details of the users or their habits. This approach was adopted to ensure that the research team could not make any pre-conceived assumptions on the configuration of data pre-processing and analysis. This proposed approach could be seen as being similar to a real-world use-case where it is not logistically possible to provide reliable labelling or annotation of the sensor data. As such, the aims of this paper are:

- To identify a minimal set of features (and learning dataset) and evaluate if this is adequate to describe a person’s daily behavioural patterns.
- To identify baseline behavioural patterns by using unsupervised self-organising algorithms such as cluster analysis, as it is not pragmatic to assume that there will be activity labels available. Clustering is an important process for providing a summary and a general overview of the data stored.
- To assess if baseline behavioural activity patterns could be used to identify users with similar behaviours by using appropriate visualisation techniques.
- To investigate if the selected set of features are able to discriminate the clinical status of users by performing an a-posteriori dialogue with the care-service provider.

The remainder of the paper is organised as follows: Section 2 summarises the most relevant related work. Section 3 and Section 4 describe the methodology and the data analysis, respectively. Section 5 reports the key results and Section 6 and Section 7 include the discussion of the findings and conclusions.

## 2. Related Work

Sensor-based monitoring technologies are becoming one of the key means to achieve a more efficient telecare system, allowing frail older adults to live independently in their home as long as possible with an improved quality of life [1]. For instance, the relationship between home-sensor based measures of everyday activities and clinical assessment has been tested over the last decade as described by Peetoom et al. [19], Cook et al. [20], and Ni et al. [21]. Kim et al. [22] compared users’ behavioural patterns within a residential facility in order to identify dementia wandering symptoms. Pavel et al. [23] observed mobility in smart homes and found evidence to support the relationship between mobility changes and cognitive decline. Dodge et al. [24] presented a study on the correlation between in-home walking speeds and variability trajectories associated with mild cognitive impairment. They used unobtrusive environmental sensors and analysed the walking speed and the time spent outside the home.

If human activities are correctly recognized, a wide range of ambient intelligent services becomes possible, such as the detection of abnormal behaviours, routine monitoring, professional advice, and data visualization for carers. According to a recent review [21] in this research field, these solutions could enhance the quality of life of the older population providing services mainly related to three main areas: “Specific health monitoring”, “Daily activity monitoring, prediction and reminding”, and “Detection of anomalous situations”. Additionally, this review underlines that important aspects related to the selection of sensors, raw data processing, and activity recognition and representation have not yet received enough attention in the real living contexts.

Therefore, the aim of this research work is to focus on the use of unlabelled low level sensory data collected as part of people’s everyday living in their homes and to extract information on their behaviour. In this context, movement detection sensors in a home environment are used to detect human activity and estimate interactions with everyday objects or identify activities performed in a specific area of the house. Gil et al. [25] defined the aggregation of movement and activity within a home as “busyness”. This measure gives an overall view or measure of people doing things without giving a detailed analysis of the individual activities that people are performing, thus preserving personal privacy [26]. The research described in this paper is based on the analysis of unlabelled movement detection sensor data only and explores the efficacy and utility of this analysis for supporting care staff.

Recently, Dawadii et al. [27] proposed a clinical assessment using an activity behaviour approach to predict cognitive and mobility scores. Their research revealed a relationship between the cognitive assessment score and the smart-home based behaviour data. However, they monitored a specific set of daily activities (six activities) and personal mobility. Since human behaviour is complex to determine and monitor reliably, these approaches could limit behavioural activities being considered what happens if a new set of activities occurs. On the contrary, Lofti et al. [28] investigated different ways of visualizing large sensor datasets and representing them by using common clustering methods to detect out-layers and abnormalities. In particular, they did not focus on specific activities, but they combined multiple sources of raw sensor data to extract the start-time and the duration of an event. However, they evaluated their approach in only two different use cases, and they did not investigate what happens if “new events” occur. Additionally, Ordóñez et al. [29] proposed an automated behaviour analysis system to assist the elderly and individuals with disabilities who live alone. In particular, they introduced three probabilistic features, namely sensor activation likelihood, sensor sequence likelihood, and sensor event duration likelihood to investigate whether their approach was able to detect anomalies, by the learning normal routine.

Skubic et al. [30] presented preliminary work on automated health alerts using unobtrusive in-home sensors. However, they generated their algorithm considering only two elderly participants and the ground truth table was generated manually by carers. Each day was labelled as a normal or abnormal day. Barger et al. [31] described a monitoring system with nine PIR sensors to infer behavioural patterns. However, their approach was validated with only one user and manual log files.

Galambos et al. [17] presented a study based on constructing an activity density map inside the house and analysing the time spent away from home to determine if there were any changes which reflected a change in people’s mental health status. However, they presented a case study with just five users, considering only the average number of events recorded within a specific time of day. Additionally, the participants’ health status was assessed by different doctors, an aspect that could influence the results. Wang et al. [32] presented a methodology for analysing data from passive infrared sensors to define an activity density map visualisation and to investigate similarity among older participants. They analysed data acquired from three different case studies to underline how these density maps could be used to track activity.

In relation to machine learning approaches for discovering behavioural patterns, supervised methods have also been widely used to recognise activity, as summarised by these recent reviews [21,33]. However, the annotation of activity is compulsory for supervised machine learning, and this requires a considerable amount of human time and effort, which makes it impractical for large data sets with numerous participants.

In this context, we propose a method to describe users’ behavioural patterns starting from unlabelled sensor data analysed with unsupervised cluster techniques. We consider 17 subjects living independently in which 100 days of sensor data from their homes was collected. In this study, we do not focus on discovering a specific activity, but we aim to generate an “activity model” considering an individual’s “busyness” metrics and profiles over different times of the day. Additionally, we investigate the potential of the features extracted from this data for distinguishing between two different groups of users using unsupervised cluster analysis and a “blind approach”.

## 3. Material and Methods

In order to achieve all the stated aims of this study, a structured methodology to the data acquisition and analysis was applied. The first part is related to the data acquisition using a “blind” approach for the data collected from 17 homes instrumented with sensors.

The second part is related to the data analysis. Our aim was to identify the similarities and differences between users and to validate with the care service provider the prediction of the affinity of a specific user to a particular “clinical group”. The key aim is to help carers better understand whether the support being provided is appropriate in relation to changing needs over time and to therefore fine-tune individual care plans.

In this section we describe the commercial IoT system used, the experimental settings, the residents, and the experimental method used to record the data. The following section describes in detail the data analysis performed.

### 3.1. Instrumentation

The commercial IoT system used was the Vera Smart Home Controller [34]. The system comprises small, wireless sensors connected to a gateway, where data is collected and sent to an online secure database. This chosen solution is “plug and play” and the sensors are FIBARO motion wireless sensors (zWave) which can be easily installed within minutes in each house without disrupting the environment [35]. We used the default option for the motion sensor’s blind time (8 s).

**Figure 1:**
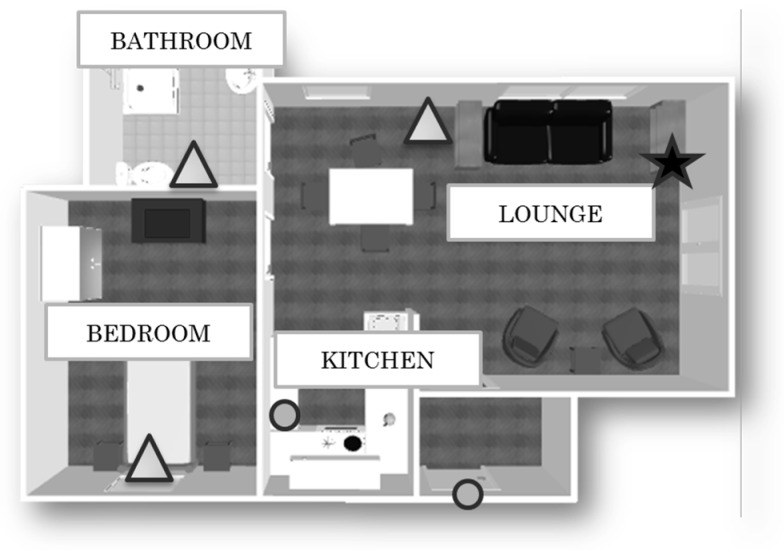
Example of the experimental set-up. The triangles are the Passive InfraRed (PIR) motion sensor, the circles are the fridge door/front door magnetic switch sensors, and the star is the gateway.

Each dwelling is instrumented with a gateway connected to the internet, and three Passive InfraRed (PIR) motion sensors, placed in the bedroom, in the lounge, and in the bathroom, which capture the resident’s movements within a specific room. Two wireless magnetic contact switch sensors are installed on the front door and on the fridge door (see Figure 1). These sensors unobtrusively and continuously monitor the resident’s activity without modifying the execution of the tasks. In the online database, sensor outputs consist of four attributes: the user ID, the timestamp, the sensor’s value, and the sensor location.

### 3.2. End-Users Participants

This study relates to 17 extra-care housing community-dwelling older adults (namely U1, U2, U3, U6, U7, U8, U9, U10, U16, U18, U41, U42, U43, U44, U45, U47, and U49) who live in sheltered accommodation in UK. All the participants filled a consent form and as part of the ethics process, all the participants’ identities were completely anonymised when made available to the researchers. As such there was no information regarding gender, age, or specific age-related impairment available at the start of the study.

The sheltered accommodation from where the data was collected is run by a local council social care provider. They support the residents by providing a care package based on a person’s specific needs. This comprises visits of one or more times a day of durations from 15 min to longer to provide support for activities of daily living as appropriate. The care planning is done on the basis of a specialist assessment and takes into consideration the person’s preferences and individual situation.

At the start of the study, the only information made available to the researchers was that the participants were from two cohorts of residents: older persons (OP) and persons with cognitive impairments (CI). Apart from this, the researchers did not have any a priori information regarding the correlation between a user’s ID and their clinical “group” (OP/CI).

### 3.3. Data Acquisition

Sensor data was gathered from 17 homes for 100 days. The participants were asked to act in a natural way and perform their daily activities as they usually do.

At the end of the data collection period, the data was retrieved from the database and analysed off-line in order to identify participants with similar behaviour patterns and to assess if the user behaviour described by the selected features could be correlated with the correct clinical group.

## 4. Data Analysis

In this section we detail all the data analysis performed to achieve all the goals of this study. The methodological approach applied within this research includes the following steps, which are detailed in the next subsections.

- Step I―includes the data pre-processing, feature extraction, and the dataset preparation, in order to explore the users’ behaviour at different Times of the Day (ToD).
- Step II―aims to identify people’s daily pattern behaviour. In particular, we investigate the minimal dataset size for obtaining an acceptable cluster model.
- Step III―includes the analysis over the night-time period (ToD1) and the validation of the cluster model with unseen data.
- Step IV―includes the visualisation of the feature space to identify similarity and diversity between users.
- Step V―includes the comparison of the different behavioural patterns in the different rooms over the selected ToDs.
- Step VI―includes validation with carers. The first part of this step aims to corroborate the correlation between the obtained clusters and the clinical cohorts.

### 4.1. Step I: Data Pre-Processing and Feature Extraction

In the first instance we considered different ways of segmenting the data in order to explore the users’ behaviour at an appropriate level of the granularity of time; in this work we do not focus on a specific activity, but rather the way the activity is distributed over a certain period of time according to the definition of “busyness” [25]. Hence we investigated different time segments, namely “*Times of the Day*” (ToD), within which to analyse user activity. In this study, we divided the day according to the time segments shown in Table 1 similar to the segmentation reported in [25].

Then, the data was retrieved from the secure cloud database and analysed off-line. In this study we consider only the three PIRs, installed in the bedroom, in the bathroom, and in the lounge (Figure 1), as our aim was to explore a minimal viable configuration of sensors in the first instance. The PIR motion sensors are used to capture the general activity levels in a home during a specific ToD period.

**Figure 2:**
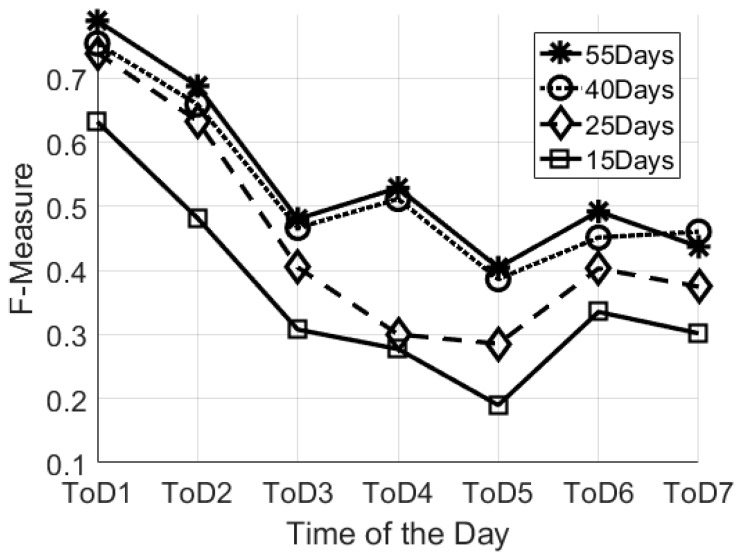
Comparison of the different learning set configurations by Means of F-Measured over different times of the day.

In this work we consider two aspects of “busyness”. The first aspect is related to the average number of specific sensor events which occur within a specific ToD (Figure 2). Our adopted approach corresponds to the one presented in [30], however we considered a higher granularity of time as described in the previous section. The second aspect is related to the temporal relationship between consecutive events. In this work we refer to this parameter as the “time between events” (TE) for a specific ToD which is computed as:

(1) $$\forall t\in TOD_{k}:\;TE=\frac{\sum_{i=1}^{m-2}\frac{\left(T_{i+2}-T_{i+1}\right)+\left(T_{i+1}-T_{i}\right)}{2}}{m}$$

where *T_i_* is the time at which a single event *i* occurs. *T_i_*, *T_i_*_+1_, and *T_i_*_+2_ belong to the same ToD and m is the total number of events over a certain ToD. A total of 42 features for each user’s dataset were computed, as summarized in Table 2. Then, the order of the days were randomized in the training and test sets, in order to reduce the influence of the weather or other external factors [36].

### 4.2. Step II: Identifying Peoples’ Daily Baseline Behavioural Patterns

In this study, five different training dataset sizes have been analysed in order to investigate the optimal number of days for obtaining a robust cluster model able to identify the participant’s behaviour over the different ToD. Specifically, for each user we consider four dataset sizes which include different number of days (78, 57, 36, and 22 days, respectively). Following this, each dataset is split into two datasets, the learning set (70%) and the test set (30%). The 7 ToD phases are used as labels for each row in the dataset.

Multiclass Support Vector Machines (SVM) developed by Mishra A. [37] and adapted by Neuburger C. [38] with a linear function for the kernel are used to group the activities.

Then, for each dataset size, a confusion matrix was created and the average F-Measure [33] was computed in order to identify the optimal trade-off between the number of days and the obtained clusters, which helped to determine the best combination and the ToD. Next, each participant’s dataset was pre-processed in order to compute the mean value and the standard deviation for a total of 84 features for the learning set and the test set (42 mean values (M) and 42 standard deviations (SD) as depicted in Table 2). Each participant is now described by a 1xF matrix in these two datasets. Thus the final dataset was described by a matrix NxF, where N is equal to the number of participants and F is the number of the features.

According to Wang et al. [32], a resident with a sedentary lifestyle could generate only 50 motion events, whereas an active person may generate more than 400 motion events per hour. Consequently, in order to estimate whether our participants lead a sedentary lifestyle or not, the mean “busyness” value was computed as the mean value over the different ToDs.

### 4.3. Step III: Cluster Analysis: Analysis of Night-Time Behaviour

**Figure 3:**
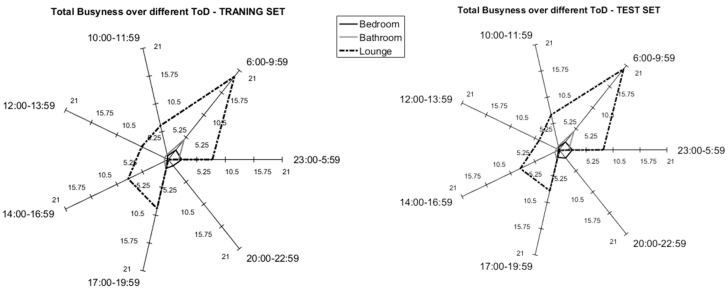
Example training set (**left**) and test set (**right**) comparison in terms of mean Busyness over the different Times of the Day (ToD) for U1.

On the basis of the previous outcome (Figure 3), we were able to determine the ToD with the highest F-Measure. In particular, in this analysis, we considered only the part of the dataset related to the ToD1 using a total of 12 features (6 M and 6 SD), as depicted in Table 2. This period of the day is selected as there is likely to be reduced noise due to the absence of external visitors (family and professional carers).

This dataset was then divided into training/test sets (55 days/23 days), according to the results gained in the previous analysis. The Pearson Coefficient (ρ) was computed for the training set to remove the most correlated features (ρ > |0.85|). Following this, Principal Components Analysis (PCA) was conducted to reduce the number of components. According to the Kaiser Rule, we considered only components with eigenvalues greater than 1.

Unsupervised machine learning clustering techniques are then used to group the participants. Particularly, in this study, K-Means (KM) and Self-Organizing Maps (SOM) are used and compared, as these methods have been shown to be the most robust [39]. Similarly, for this reason, the Dunn’s index [40] and Silhouette index [41] are computed to assess the cluster validity and to quantitatively analyse the clustering process. A higher value for both indexes indicates that the clusters are compact and well separated. Then, the performance of the cluster models was estimated. The two cluster models previously constructed (KM, SOM) are tested with new and previously unseen data (Test Set).

Firstly, PCA is applied to reduce the test set features. Thereafter, using KM, the centroids of each class, identified with the training set, were used to estimate the group to which a participant belonged (considering the test set) using a measure of the closeness to the cluster centre. Similarly, this analysis was performed using SOM as well, where the trained net was tested with new data. Then, the new clusters were compared with the results obtained with the training set.

### 4.4. Step IV: Identifying Users with Similar Daily Behaviour Patterns

The complete training dataset configuration (84 features) was analysed in order to map the feature space over a full 24-h time period. For this analysis, two different methods were used to identify participants with similar behaviour patterns. Firstly, a preliminary visual inspection of the data was performed to identify the outliers and any groupings of similar participants. Then KM and SOM were applied to group the users according to their daily behaviour patterns.

Firstly, ρ was used to remove the most correlated features (ρ>|0.85|); PCA and Sammon’s Mapping (SM) [42] have been established as efficient approaches for the two-dimensional representation of data. Particularly, as presented in [43], both approaches are applied to reduce the number of features and improve the visualisation of the feature space. Our aim in selecting these methods was to use a data representation format that carers would find easier to understand and would thus provide feedback (see Section 4.6).

Finally, a Similarity Matrix was computed to explore the relationship between points on the SM and to quantify the “similar behaviour” in the dataset. The equation of the similarity matrix (*Simi*) is based on the normalized Euclidian distance and is computed as:

∀A(x,y),B(x,y)∈SM;

(2) $$ED_{A,B}=\sqrt{{\left(X_{A}-X_{B}\right)}^{2}+{\left(Y_{A}-Y_{B}\right)}^{2}}$$

where *A* and *B* are two points of the *SM*. The ED matrix comprises the Euclidian distance (ed) values computed between all the points. A generic element (*k*th) of Simi is computed as:

(3) $$Simi_{k}=\;\left|\frac{ED_{i}-min\left(ED\right)}{max\left(ED\right)-min\left(ED\right)}\right|$$

where $ED_{i}$ is the ith element of the ED matrix, and min(ED) and max(ED) are the minimum and the maximum value, respectively. Simi’s values fall between 0 and 1.

### 4.5. Step V: Comparison of Daily Behaviour Patterns in Different Rooms

In order to corroborate the previous results, this analysis was aimed at identifying any similarities or differences in participants’ behavioural patterns considering each room separately, as an independent “block”. The complete dataset (described in the previous sections) was divided into three sets, one for each room. Each dataset was thus reduced to 28 features (Table 2). As previously described, ρ had been computed to remove correlated features, and PCA and SM were used to allow a two-dimensional representation of the feature space. Additionally, KM and SOM were used to group participants with similar behaviour in each of the rooms (Bedroom, Bathroom, and Lounge).

### 4.6. Step VI: Validation with Carers and Therapists

All the analyses described in the previous sections were conducted in an anonymised manner as we did not have any information about the participants. Subsequent to the cluster analysis, we obtained information from the care service provider regarding the study participants’ clinical status and habits to corroborate the previous cluster analysis. We wanted to validate any correlations between participants falling in specific clusters and belonging to a particular clinical group (OP or CI) as they had been designated. All the information was acquired through an email dialogue on sharing the results achieved in the previous sections.

All the clusters (Night-time, 24 h, 24 h-Bedroom, 24 h-Bathroom, and 24 h-Lounge) were compared with the real clinical cohorts as reported by the care service provider. The results in the next section are presented as a confusion matrix. Then, overall Accuracy, Specificity, and Sensitivity metrics have been calculated to estimate the effectiveness of the models [33] and to compare their performance.

## 5. Results

This study had a number of objectives. Firstly, it sought to evaluate if the selected features related to the “busyness” measure could be used to identify the participant’s daily behaviour patterns. To achieve this, we also needed to validate the results with the care service provider in order to evaluate the efficacy of our clustering approaches. In this section, we are going to present the results as per these objectives.

### 5.1. Minimal Number of Days Needed to Establish Baseline User Behaviour Patterns

As described in the previous section, we varied the learning sets using different numbers of days (55, 40, 25, 15 days). Figure 2 reports the average F-Measure over the different Times of the Day. The high value of F-Measure is for the “55-days” configuration for the training set. It is worth mentioning that the highest values for F-Measure correspond to the night-time (ToD1–0.7896), whereas the lowest values correspond to the central part of the day (0.4804 and 0.4045 for ToD3 and ToD5, respectively). This confirms that during the central part of the day there is a lot of variability in the behaviour, which is not easy to identify with unannotated data. Indeed, this trend is confirmed by results obtained from all the different dataset sizes. However, the “planned” visits (i.e., care-package or routine visits from family members) could be considered as systematic noise in the system, and could be seen as being an integral part of the individual’s behaviour pattern. During the night we have no external visits and no visits from professional carers, thus we can consider this time period as the optimal configuration to estimate the baseline behaviour. It is important to notice also that the “40 days” configuration gives similar results, however, in this study, we decided to focus on the “55-days” configuration for the training set because it has good results for all the considered ToD. According to the methodology described in the previous section, the “55 days” configuration means a total of 78 days analysed: 55 days for the training of the models and 23 days to test the models with unseen data.

Additionally, analysing the training set, the participants generated an average “busyness” value of 128 events (standard deviation: 47 events, min: 61 events and max: 215 events). It is worth mentioning that the results of the test set showed comparable values; in effect, the average busyness was equal to 128 (standard deviation: 45 events, min: 67 events and max: 201 events). Figure 3 reports the mean activity for the training set (55-days) and the test set (23-days) of participant U1 as an example. These results are aligned with the literature as reported by Wang et al. [32]

### 5.2. Minimal Number of Days Needed to Establish Baseline User Behaviour Patterns

**Figure 4:**
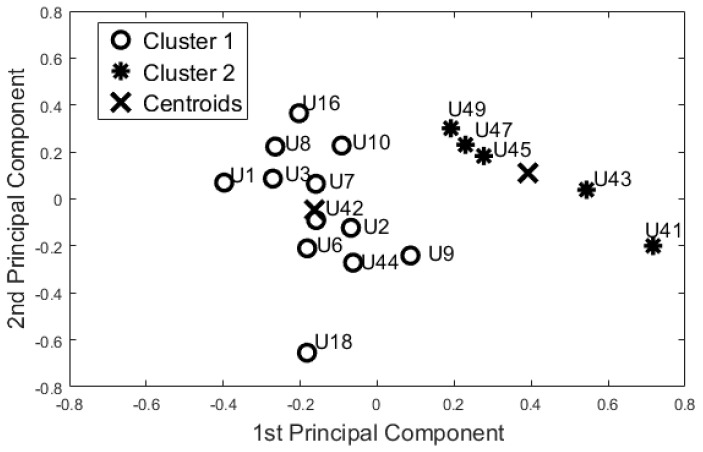
Night-time Period - Cluster Analysis and K-Means (KM) Centroids obtained with the Training Dataset “55-days”.

On the basis of the results for identifying baseline user behaviour (Section 5.1), we decided to focus only on the 55-days configuration. The 12 features used in the dataset were uncorrelated (ρ<|0.85|). According to the Kaiser Rule, we reduced the learning dataset to four components. The cluster methods were applied on this 4-component dataset. Both algorithms showed the same output. Dunn’s index was equal to 0.310, whereas the Silhouette index was equal to 0.473 for both methods. Figure 4 shows the space of the first two principal components (~61% of the information).

The evaluation of the cluster models was performed with the test set. The test set comprised 23 unseen days, and both the algorithms clustered the participants in the same group as the learning set. These results suggest that the selected features are able to produce repeatable clusters, representative of the participants’ behaviour patterns.

### 5.3. Minimal Number of Days Needed to Establish Baseline User Behaviour Patterns

In order to investigate the ability of this set of features to capture the similarity between participants over a 24 h period, the complete dataset (84 features) was reduced using the Pearson Coefficient to 65 features. For the data from the bedroom, we thus removed the busyness mean value for ToD2, ToD3, ToD4, and ToD7; and the busyness SD values for ToD2, ToD5, and ToD6. We also removed the TE mean values for ToD4. For the data from the bathroom, we removed the mean busyness values for ToD4 and the busyness SD for ToD2, ToD3, and ToD6. For the data from the lounge, we removed the busyness mean values for ToD3 and the busyness SD for ToD1, ToD4, ToD5, and ToD7. The mean TE values for ToD6 and ToD7 were also removed.

**Figure 5:**
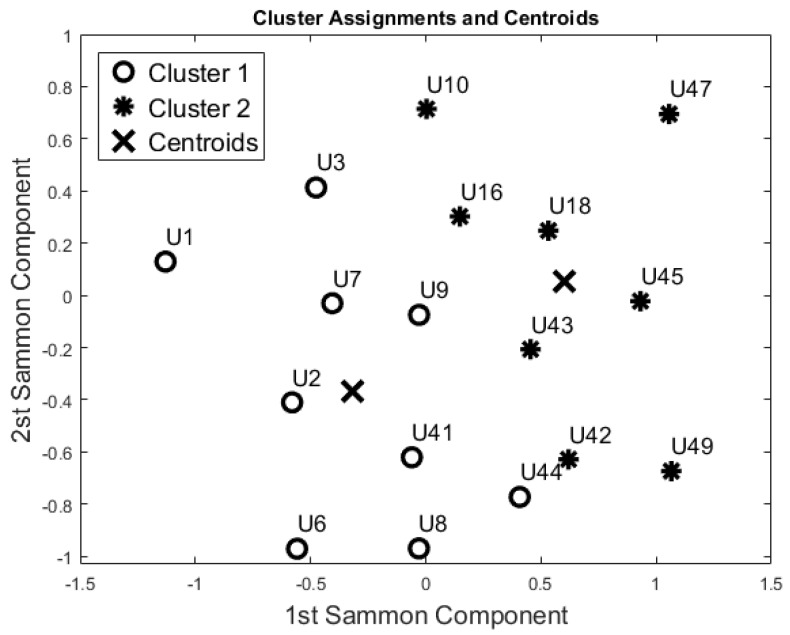
Cluster Analysis on the 24-h period performed on the “55-days” training set. Both unsupervised methods gave similar results.

Then, PCA and SM were applied to this reduced dataset to visualise the feature space. According to the Kaiser Rule, we reduced all the learning datasets to 14 components, and with the SM techniques we also reduced the dataset to a bi-dimensional problem (from 14 components to 2 components). Both unsupervised methods showed the same cluster groupings as depicted in Figure 5. The Dunn’s index was equal to 0.196, whereas the Silhouette index was equal to 0.469 for both algorithms. Comparing these results with the Night-time analysis (Figure 4), participants U44, U42, U18, U10, U16, and U41 were grouped in a different cohort. The other participants were classified in the same cohort as the night-time analysis.

From a visual inspection of the SM (Figure 5), participant U1 is further away from the other participants. Particularly, participants U1 and U49 (ED = 2.332) are the furthest away. The mutually closest points on the map are participants U42 and U44 (ED = 0.250). These two ED values have been used as the maximum value in the computation of the Simi matrix. The closest points with *Simi* < 0.05 are participants U18 and U8 (*Simi* = 0.023). Other points closer to Simi are between 0.051 and 0.1, which are U41 and U18 (*Simi* = 0.051), U7 and U9 (*Simi* = 0.057), U3 and U7 (*Simi* = 0.080), U9 and U16 (*Simi* = 0.082), U42 and U43 (*Simi* = 0.082), U16 and U43 (*Simi* = 0.086), U45 and U43 (*Simi* = 0.089), and U2 and U7 (*Simi* = 0.097). The Simi matrix confirms that participant U1 is the most distant point in the feature space.

### 5.4. Minimal Number of Days Needed to Establish Baseline User Behaviour Patterns

**Figure 6:**
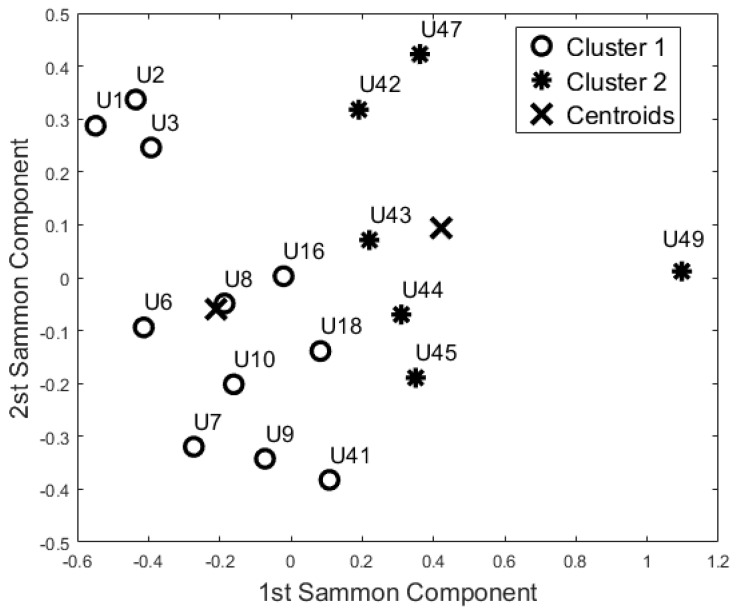
Cluster analysis of Bedroom Activity (K-Means and Self-Organizing Maps).

**Figure 7:**
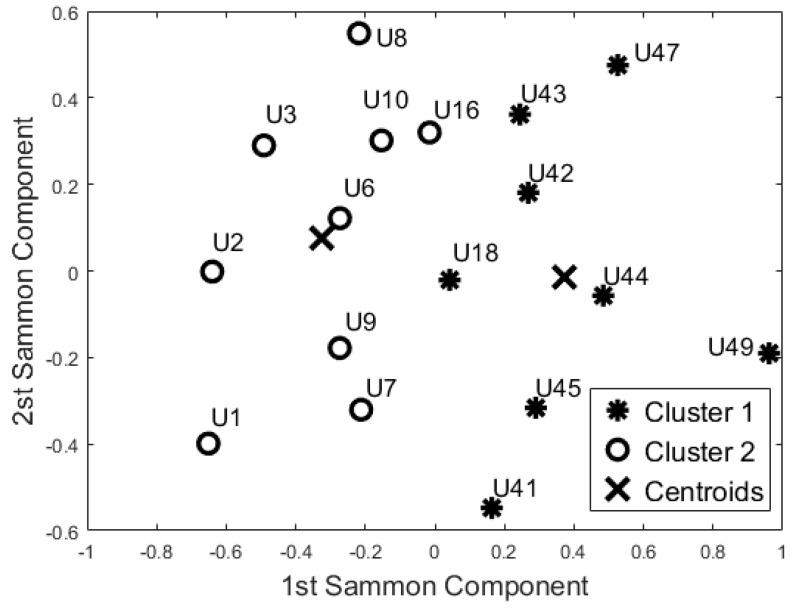
Cluster analysis of Bathroom Activity (K-Means and Self-Organizing Maps).

**Figure 8:**
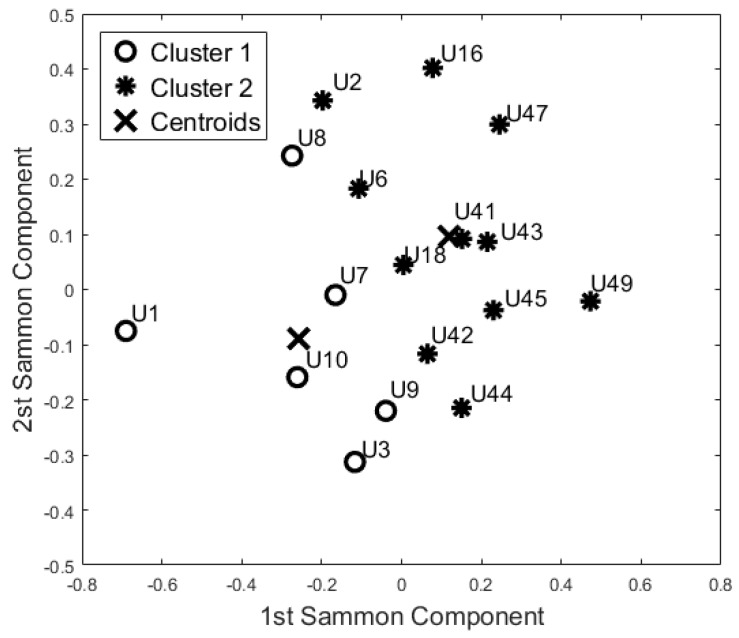
Cluster analysis of Lounge Activity (K-Means and Self-Organizing Maps).

The aim of this analysis is to investigate whether the sensors are able to group the participants according to their busyness patterns. In particular, we performed three additional analyses, considering each room as a separate block, to verify if the participant has a comparable activity pattern over the different rooms and if they could be grouped by similarity. The results of these analyses from the three rooms are reported in Figure 6, Figure 7 and Figure 8.

According to the Pearson coefficient, we reduced the 24 h-bedroom dataset to 19 uncorrelated features (the mean busyness for ToD3, ToD4, ToD5, and ToD7, busyness SD for ToD2, ToD4, and ToD6, TE mean values for ToD5, and TE SD for ToD4 were not included); the 24 h-bathroom dataset was reduced to 24 features (the Mean Busyness for ToD4, Busyness SD for ToD2, ToD3, and ToD6 were not included) and the 24 h-lounge dataset reduced to 21 features (the mean busyness for ToD3, busyness SD for ToD1, ToD4 and ToD5, TE mean values for ToD1, and TE SD for ToD6 and ToD7 were not included). Then, according to the Kaiser rule, we reduced the 24 h-Bedroom to four components, the 24 h-lounge dataset to a five component dataset, and the 24 h-bathroom dataset was reduced to a five component dataset. Both unsupervised algorithms generated the same clusters using this data.

The cluster analysis of participants’ activity in the bathroom (Figure 7) and in the bedroom (Figure 6) generated the same two groups (except for U18) even though the distribution of the points in the SM space was different. With respect to the night-time analysis, the bedroom activity classifies U41 and U42 in two different cohorts, whereas the other participants belong to the same cohort. On the contrary, the bathroom activity analysis revealed a different cohort for U42 and U44. From the analysis of the lounge activity, U2 and U16 were classified in a different cohort (Figure 8).

### 5.5. A-Posteriori Validation with the Care Service Provider

In this study we used a “blind” approach as previously stated, and we did not have any a priori knowledge about the participants’ clinical assessments, habits, gender, or any other personal information. At the end of the data analysis, we presented all the results to the care service provider in order to compare the outcomes with their knowledge of the participants concerning their health status and clinical status. According to their classification, the participants could be classified into the following two groups according to their clinical status:

(Older Person) OP= {U1, U2, U3, U6, U7, U8, U9, U10, U16, U18}(Cognitive Impairment)CI={ U41, U42, U43, U44, U45, U47, U49}

The comparison between our cluster analysis and the carers’ feedback gave a high correspondence (>80%), revealing the clustering of clinically similar participants. Particularly, the overall accuracy is equal to 0.88 for the night-time period, 0.94 for the 24 h period and the 24 h-bedroom, 1.00 for the 24 h-bathroom, and 0.76 for the 24 h-lounge activity. The two cluster algorithms have similar results for all the cluster analyses performed and thus they can be presented and discussed together.

For the night-time period, 10 participants out of 10 are correctly classified as OP (100% of the total), whereas 5 participants out of 7 are correctly assigned to the CI cohort. With regards to the sensitivity metrics, it is equal to 0.83 for the OP cohort (True Positive). Particularly, out of the 12 OP predictions, 83% are correct, whereas out of the 5 CI predictions, 100% are correct.

With regards to the 24 h analysis, 7 participants out of 10 are correctly classified as OP (True Positive), whereas 7 participants out of 7 are correctly classified as CI (True Negative). The overall accuracy is equal to 0.94. For the 24 h-bedroom analysis, 10 participants out of 10 are correctly classified as OP, whereas 6 participants out of 7 are correctly classified as CI. In this case the sensitivity is equal to 1.00 and the specificity is equal to 0.86. For the 24 h-bathroom analysis, the participants were correctly classified as OP and CI. With regards to the 24 h-lounge dataset results, 8 participants out of 10 were correctly classified as OP and 7 participants out of 7 were correctly classified as CI. The complete results are reported in Table 3.

## 6. Discussion

In this paper we present a method based on a “busyness” measure to estimate participant behaviour without focusing on a specific set of activities. Our approach aims to verify whether it is possible to reveal similarities and differences by using an easily generalisable approach that does not require labelled data and therefore does not require extensive effort from care staff in setting up and configuring the system.

One of the big challenges in the field of activity recognition is deriving a minimum “set of features” to perform recognition tasks with a high level of accuracy [33]. In this study we found that 55 days was adequate to construct a learning set; in effect, the evaluation of the clusters formed using this dataset confirms that the resulting models are robust with unseen data (F-Measure 0.80). Additionally, the night-time period (ToD1) was found to be adequate in capturing baseline behaviour patterns. In this period there are reduced “external” disturbances under normal conditions, no visits from carers or from family members, as confirmed by care service provider. This means that users with similarities in their care packages (U42, U18, U10, and U9) are distant in the map (Figure 4), whereas they would not be if the day-time data was used. Additionally, based on the information provided by the care service provider, participants were classified in the two clinical cohorts they had identified with a high degree of accuracy (>90%). In particular, the accuracy seems to increase if we consider each room separately (0.94 for the bedroom and 1.00 for bathroom data).

Additionally, the results of this study suggest that the proposed “blind” approach could be reliably used to group and distinguish participants with similar behaviour. For instance, participant U1 could be seen as having a different behaviour pattern over the 24 h period in comparison to the other participants (Figure 5, Figure 6, Figure 7 and Figure 8). The reported information from the care service provider confirmed that participant U1 does indeed have quite different habits; he is one of the least active older residents and also receives very little care. Additionally, as depicted in Figure 5, participant U9 is on the edge between the two cohorts. The care service provider reported that while this participant belonged to the OP cohort, they had more aspects in common with the CI cohort, which is why both the cluster algorithms seemingly “wrongly” classified this participant. From the carer’s reports, this participant receives a lot of visits from her family who regularly come to visit. It is worth mentioning that for the night-time analysis, participant U9 was not on the edge between the two cohorts; this suggests that the night-time analysis could be used to measure the busyness of single participants and thus exclude multi-occupancy noise. Furthermore, participant U18 is on the edge of the cluster in the room analysis results (Figure 6, Figure 7 and Figure 8); this participant also seems to have more features in common with both groups. In fact, the results show that U18, who also belongs to the OP cohort, is classified as belonging to the CI group in the 24 h analysis (Figure 5). The care service provider confirmed this finding, reporting that U18 has fairly advanced dementia.

Participant U44 has been classified in the OP cohort in the Night-Time analysis, and he seems to have fewer characteristics in common with the CI cohort. The dialogue with the care service provider revealed that U44 keeps very structured routines. He goes to bed early and he is out of his flat regularly during the day. Findings and results like these discovered by our clustering approaches can be used by care service providers to improve the discussion with the participants about their daily routines and could lead to the prevention of dangerous situations or improvements to their care planning, making sure that the best possible personalised care package is delivered.

It is worth mentioning that U1, U3, U6, U7, and U9 were always classified in the same cohort, as well as U49, U45, U43, and U47. The patterns of these participants are quite different as shown by the visualisation of the feature space. However, they were always grouped into the same cluster. Carers stated that the first group of participants belonged to the OP cohort, and the other participants were designated as belonging to the CI group.

Future analysis should include an inter-subject approach in order to evaluate if these features are able to distinguish variance between individuals.

## 7. Conclusions

This paper presents our findings that illustrate how unlabelled movement sensor data from a reduced number of low-cost commercial sensors can be used to develop a “busyness” set of features, which enable the recognition of users’ behaviour patterns over a short learning period. In particular, we propose an unsupervised learning based “blind” approach which could be seen as being reflective of a real-world use-case where it is not logistically possible to provide reliable labelling or annotation of the sensor data. In this study, environmental data from 17 community-dwellers were analysed in order to provide information on their behaviour patterns, to investigate correlations with the clinical cohort to which they had been allocated, and to identify similarities between individual residents.

The results obtained suggest that the features we derived in this study are able to group participants with similar behaviours and discriminate between those that do not have similar behaviours. Furthermore, the proposed approach is also able to identify participants with specific behaviour patterns and profiles that have the potential to inform more personalised caregiving and support. The approach described in this study could also be used to track participants’ needs over time and fine-tune a person’s care plan, optimising the care process.

## Figures and Tables

**Table 1:** Segmentation of day-time (24 h) into time segments (*Times of the Day*).

Phase	Time
Night Time	23:00–5:59
Early Morning	6:00–9:59
Late Morning	10:00–11:59
Early Afternoon	12:00–13:59
Afternoon	14:00–16:59
Evening	17:00–19:59
Late Evening	20:00–22:59

**Table 2:** Summary of features used in the analysis. M is the mean values and SD is the standard deviation computed for a specific “Times of the Day” ToD, whereas TE is the “Time between events”.

Features	Learning Set’s Size	Night-Time	24 h	24 h Bedroom	24 h Bathroom	24 h Lounge
Bedroom Busyness	7 M	1 M + 1 SD	7 M + 7 SD	7 M + 7 SD	-	-
Bedroom TE	7 M	1 M + 1 SD	7 M + 7 SD	7 M + 7 SD	-	-
Bathroom Busyness	7 M	1 M + 1 SD	7 M + 7 SD	-	7 M + 7 SD	-
Bathroom TE	7 M	1 M + 1 SD	7 M + 7 SD	-	7 M + 7 SD	-
Lounge Busyness	7 M	1 M + 1 SD	7 M + 7 SD	-	-	7 M + 7 SD
Lounge TE	7 M	1 M + 1 SD	7 M + 7 SD	-	-	7 M + 7 SD
Total Features	42	12	84	28	28	28

**Table 3:** Validation with carers. Comparison with clinical cohort.

Analysis	Cluster	Sensitivity	Specificity	Accuracy
Night-Time	KM	0.83	1.00	0.88
	SOM	0.83	1.00	0.88
24 h	KM	1.00	0.88	0.94
	SOM	1.00	0.88	0.94
24 h Bedroom	KM	1.00	0.86	0.94
	SOM	1.00	0.86	0.94
24 h Bathroom	KM	1.00	1.00	1.00
	SOM	1.00	1.00	1.00
24 h Lounge	KM	0.80	0.71	0.76
	SOM	0.80	0.71	0.76
