# Correction: Fiorini, L., et al., Unsupervised Machine Learning for Developing Personalised Behaviour Models Using Activity Data. *Sensors* 2017, *17*, 1034

**DOI:** 10.3390/s19224860

**Published:** 2019-11-07

**Authors:** Laura Fiorini, Filippo Cavallo, Paolo Dario, Alexandra Eavis, Praminda Caleb-Solly

**Affiliations:** 1 The BioRobotics Institute, Scuola Superiore Sant’Anna, Pontedera, 56025 Pisa, Italy; filippo.cavallo@santannapisa.it (F.C.); paolo.dario@santannapisa.it (P.D.); 2 Alcove Limited, 44 Westbridge Road, London SW11 3PW, UK; alexandra.eavis@youralcove.com; 3 Bristol Robotics Laboratory, University of West England, Bristol BS16 1QY, UK; Praminda.Caleb-solly@uwe.ac.uk

**Keywords:** behavioural models, unsupervised machine learning, cognitive health assessment, real-home settings

## Abstract

A correction is presented to correct the section headings of Sections 5.1, 5.2, and 5.3 in [*Sensors*, **2017**, 17, 1034].

The authors wish to make the following corrections to this paper [1]:

The section headings in Sections 5.1, 5.2, and 5.3 are the same. The correct section headings are as follows:

*5.2. Cluster Analysis: Analysis of Night-Time Behavior*

*5.3. Analysing Similarity between Participants’ Behavior*

*5.4. Similarity between Participants over the Three Rooms*

The authors would like to apologize for any inconvenience caused to the readers by these changes.
